# Modeling of Energy Demand and Savings Associated with the Use of Epoxy-Phase Change Material Formulations

**DOI:** 10.3390/ma13030639

**Published:** 2020-01-31

**Authors:** Elena Arce, Richa Agrawal, Andrés Suárez, Lara Febrero, Claudia C. Luhrs

**Affiliations:** 1 University Defense Center, Spanish Naval Academy, 36920 Marin, Spain; asuarez@cud.uvigo.es (A.S.); lfebrero@cud.uvigo.es (L.F.); 2 Department of Mechanical and Aerospace Engineering, Naval Postgraduate School, Monterey, CA 93943, USA; richa.agrawal.in@nps.edu

**Keywords:** phase change material, epoxy composite, thermal energy storage, simulation, shipping container

## Abstract

This manuscript integrates the experimental findings of recently developed epoxy-phase change material (PCM) formulations with modeling efforts aimed to determine the energy demands and savings derived from their use. The basic PCM system employed was composed of an epoxy resin, a thickening agent, and nonadecane, where the latter was the hydrocarbon undergoing the phase transformation. Carbon nanofibers (CNF) and boron nitride (BN) particulates were used as heat flow enhancers. The thermal conductivities, densities, and latent heat determined in laboratory settings were introduced in a model that calculated, using EnergyPlus software, the energy demands, savings and temperature profiles of the interior and the walls of a shelter for six different locations on Earth. A shipping container was utilized as exemplary dwelling. Results indicated that all the epoxy-PCM formulations had a positive impact on the total energy savings (between 16% and 23%) for the locations selected. The use of CNF and BN showed an increase in performance when compared with the formulation with no thermal filler additives. The formulations selected showed great potential to reduce the energy demands, increase savings, and result in more adequate temperatures for living and storage spaces applications.

## 1. Introduction

Thermal energy storage (TES) systems, such as phase change materials (PCM), can create a balance between day and night energy demands by storing thermal energy in the form of latent heat [1,2]. PCMs, due to their high latent heat of fusion, stand out from other TES due to their ability to store larger amounts of energy within small temperature intervals [3]. Therefore, PCMs can be an attractive solution to improve energy efficiency and thermal comfort in buildings, storage and other living spaces [4].

Depending upon the type of material, PCMs can be categorized into organic, inorganic, and eutectic subtypes. Among organic PCMs, alkanes (C_n_H_2n+2_) or paraffins have been widely studied, given their chemical stability, high latent heat of fusion, cost-effectiveness, non-corrosive nature, and compatibility of metallic containers [5]. However, one of the shortcomings of paraffins is their low thermal conductivity, which can adversely affect their energy absorption/release rates. In order to improve the thermal conductivity, conductive fillers inducing metals [6], metallic foams [7], nanostructured carbon including carbon nanofibers (CNFs) [8], carbon nanotubes (CNTs) [8], boron nitride (BN) [8], as well as boron nitride nanotubes (BNNTs) [8] have been investigated. CNFs are especially attractive owing to their high aspect ratios, which can offer significant improvement in conductivity [9], whereas BN is electrically insulating and thermally conducting [10].

PCMs, as passive heating and cooling systems, can be incorporated into building materials in different ways: gypsum boards with microencapsulated paraffin, concrete with microencapsulated paraffin, paraffin panels, bricks with PCM or wood with PCM [11,12,13]. However, once incorporated into a building, these approaches are difficult to modify or retrofit, making it necessary to develop more flexible technologies that could be incorporated into different stages of the life cycle of a dwelling.

A large proportion of shipping containers are reused in applications not linked only to the transportation of goods [14,15]. Due to their low weight, quick assembly, low cost, and versatility, portable containers are also currently used in a wide variety of applications, from housing after natural disasters to military operations. There is a large market that exploits diverse construction techniques, offering solutions ranging from single-family homes to blocks of different floors or offices [16]. Within the armed forces, maritime shipping containers have been used as field hospitals, ammunition warehouses, sanitary warehouses and drinking water treatment plants, among others [17,18]. This offers a huge capacity for transporting large volumes of humanitarian aid or facilities in a short period of time. One of the shortcomings with shipping containers is the overheating resulting from high internal heat gains and solar radiation [19], thus opening opportunities for the use of PCM that can be incorporated after the containers have been built.

Space heating or cooling consumption in developed countries accounts for half of the energy use in buildings [20]. Several studies have demonstrated that the envelope of a building influences thermal demand significantly. The envelope construction has great impact in heating and cooling demands and its optimization provides considerable benefits in terms of energy savings and increases energy efficiency of a building [21,22,23,24]. The envelope implementation of materials with high heat capacity is an effective way to generate energy efficient buildings [25]. However, when it comes to lightweight portable dwellings, such as shipping containers, using traditional materials such as blocks or stone is not an option.

For the present work, n-nonadecane (C_19_H_40_) was utilized as the organic PCM material given its melting and solidification temperatures being close to environmental conditions [8]. CNFs and BN were incorporated as the thermal conductivity enhancers as established in our previous work [8]. To prevent paraffin leakage, an epoxy resin was utilized as the support matrix material [6,8], and a thickening agent, Carbopol, was used to mitigate phase separation during sample formulation [8]. A study of energy savings in EnergyPlus [26] was carried out, in which different insulation alternatives were evaluated. The results offered in this work make it possible to establish this particular formulation and modeling strategy as a viable alternative for applications where portable epoxy-PCM technologies are needed.

## 2. Methods

### 2.1. Experimental

In order to synthesize epoxy-PCM-filler composites, determine densities, thermal conductivities and study their specific heat capacity and temperature profiles, the methodology described in the following sub-sections was adopted. Since the integration of the formulations into removable liners is proposed in later sections, we also addressed here how the composites were attached to fabric substrates.

#### 2.1.1. Synthesis of Epoxy-PCM-Filler Composite Formulations

The fabrication steps for the formulations employed and the rationale for their use can be found elsewhere [8]. The PCM (n-Nonadecane, Sigma-Aldrich, St. Louis, MO, United States) was melted using a water bath and then mixed with Part A (resin) of EpoFix (Struers Inc., Cleveland, OH, United States) using a dual asymmetric speed mixer (Flacktek, Landrum, SC, United States). The thickening agent (Carbopol, Sigma-Aldrich, St. Louis, MO, United States) was then added, followed by the thermally conducting fillers, carbon nanofibers (CNF, 2 wt.%) and boron nitride (BN, 10 wt.%) particles, both of which were procured from Sigma Aldrich. Part B (hardener) of the EpoFix was finally added to the mixture, and the formulation was left to cure at room temperature in flexiform molds for 24 h (Struers, Inc. Cleveland, OH, United States). The samples containing CNF and BN powders were designated as EC-PCM40-CNF2 and EC-PCM40-BN10, respectively. The sample preparation steps for the specimen without thermally conductive fillers, designated as EC-PCM40, were identical than for the other formulations except the filler addition. Detailed sample formulations are presented in Table 1.

#### 2.1.2. Specific Heat Capacity Measurements of the Epoxy-PCM-Filler Composites

Specific heat (Cp) measurements on the EC-PCM40, EC-PCM40-BN10, and EC-PCM40-CNF2 samples were performed using a Netzsch STA 449 F3 Jupiter simultaneous thermogravimetric analysis (STA) instrument. The experiments were performed in inert atmospheres (Ar) and the samples were placed in PtRh crucibles fitted with Al_2_O_3_ liners to achieve the highest measurement sensitivity while preventing crucibles reaction with PCM formulations. Given the melting point of n-nonadecane, the temperature range for the measurements was kept at 25–65 °C. After an initial isothermal step of 10 min at 25 °C, the samples were heated to 65 °C at a heating ramp of 5 °C/min and then finally allowed to cool down to room temperature. The Cp values were calculated by the software program Proteus employing the ratio method. Standard sapphire disks were employed as the reference material (6 mm diameter, 0.5 or 0.75 mm thickness and 3.98 g/cm^3^ density) acquired from the STA manufacturer (Netzsch, Selb, Bavaria). The typical sample mass was between 10 and 20 mg.

#### 2.1.3. Density Measurements

The density of the epoxy-PCM composites were determined by dividing the mass of the respective composites by their volumes. The mass determination was conducted using a high precision balance (Explorer® Pro, OHAUS, Parsippany, NJ, United States) and the volumes of the cylindrical composites were both, calculated using V = π·r^2^·h, where r and h are the radius and height of the cured sample, and corroborated by water immersion.

#### 2.1.4. Thermal Conductivity Measurements

Thermal conductivity measurements were carried out using a C-Therm TCi Thermal Conductivity Analyzer using the modified transient plane source (MTPS) configuration. Thermal conductivity measurements were performed at room temperature, the service was contracted with Thermal Analysis Labs (Fredericton, New Brunswick, Canada) [8].

#### 2.1.5. Epoxy-PCM-Filler Composite Integration onto Fabric and Thermal Imaging

**Figure 1:**
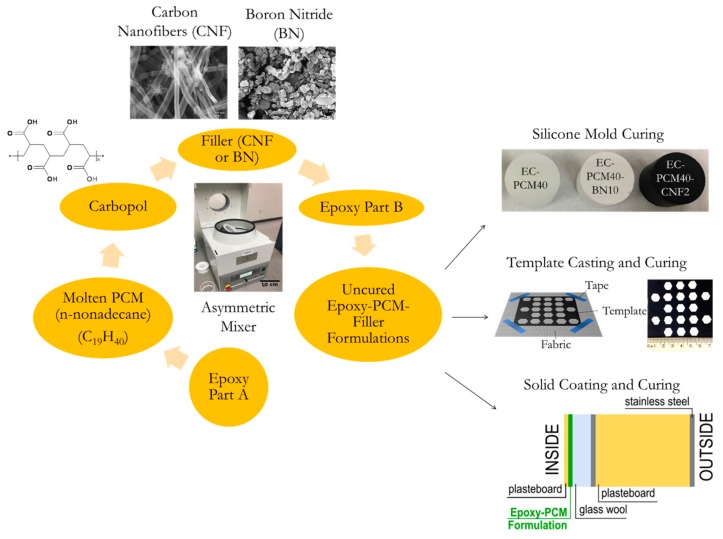
A schematic illustration depicting the process flow for silicone mold cured, template casted, as well as solid-coated epoxy-PCM-filler composite formulations.

Epoxy-PCM-filler composites were patterned on Nylon fabric using template casting. The templates were prepared on transparency films. The geometric patterns were generated using CorelDRAW software and the templates were prepared using a laser-cutter (Spirit GLS GCC, Walnut, CA, United States) The epoxy-PCM-filler formulations were prepared as described in Section 2.1.1. The uncured formulation was poured onto the template fixated on the Nylon fabric with a tape. After pouring, the formulation was spread evenly onto the fabric using a glass slide, followed by removing the template from the fabric. The patterned samples were then left to cure at room temperature for 24 h. The schematic for the process flow and representative samples for the 10 wt.% BN formulation (EC-PCM40-BN10) are shown in Figure 1. Thermal imaging on the template-casted composite samples was performed using a sand bath setup and a FLIR ETS320 infrared camera.

### 2.2. Modelling and Simulation

Thermal simulations were employed to evaluate PCM performance in building energy demands [27]. Several modeling tools have been used to address this issue. EnergyPlus [26] and TRNSYS [28] are common software utilized for modeling systems in order to corroborate experimental findings.

**Figure 2:**
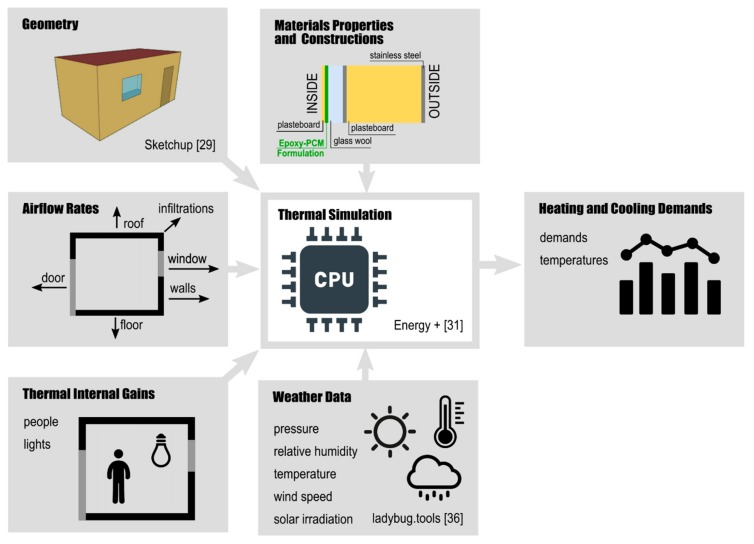
Modeling and simulation inputs and outputs workflow.

The Cp curves, thermal conductivities, densities, peak transition temperatures, and latent heat values were determined experimentally, as described above, and used for the modelling and simulation that follows. The simulation used a shipping container as an example of a lightweight portable dwelling to calculate the heating and cooling demands, envelope temperatures and energy savings for 6 different locations. The outcome of modeling the three different PCM formulations listed in Table 1 were compared to a reference container that did not contain a PCM layer. The container geometry was defined in SketchUp [29]. Thermal conditions of the simulation were defined in EnergyPlus as described in the sections that follow. Figure 2 shows the inputs used for thermal model, the analyzed outputs and each of the software programs employed.

#### 2.2.1. Dwelling Model

A generic container that included the most common construction materials and module sizes used in commercial shipping was used as reference model. It was assumed that the shipping container will be used as a simple shelter, thus it did not include subdivisions, restrooms, or power for extra equipment, similar to the configurations used in humanitarian disaster relief. The structure was designed based on the specific NATO (North Atlantic Treaty Organization) regulations for this type of construction [30]. The single-zone container (Figure 2) was a parallelepiped of 6.05 m long by 2.9 m wide and 2.85 m high. In the south facade, it contained a door (2.15 m^2^) and a window (1.13 m^2^) and in the north facade, it consisted of two windows of different sizes, 0.25 m^2^ and 0.95 m^2^, respectively. The enclosure details of the module and the thermophysical properties of the construction materials are given in Table 2. The windows had a 3 mm thick thermopanel glass. Thermal conductivity of glazing was fixed in 0.9 W/m/K. To stabilize the frame, 1 mm thick aluminum profiles were included along the contour. The glazing materials were not relevant for the thermal simulation because most of the energy transmitted through windows was assumed to occur by radiation [23].

Reference model without the PCM layer, was compared with other three PCM formulations (EC-PCM40, EC-PCM40-BN10, and EC-PCM40-CNF2) included in the external walls and roof construction. The PCM was added in the interior side of the enclosure as described in Figure 2.

#### 2.2.2. Thermal Model

Thermal simulations were performed using the dynamic building simulation software EnergyPlus (v9.1, U.S. Department of Energy’s (DOE), Washington DC, WA, USA). The total heating and cooling demands were calculated based on ideal free floating conditions, that is, it was assumed that the module had unlimited thermal resources.

The conduction finite difference (CondFD) solution algorithm was used as the heat balance algorithm because the container model included a PCM layer with variable thermal conductivity. CondFD algorithm applies an implicit finite difference scheme with an enthalpy-temperature function to account for PCM energy, that is, the container envelope is divided into nodes, and node enthalpies get updated with each iteration. Fully implicit first order (Adams-Moulton solution approach) scheme was chosen. The EnergyPlus PCM model (CondFD solution and PCM algorithms) was validated against previous research [31,32]. TARP (Thermal Analysis Research Program) algorithm was used to calculate the interior heat transfer coefficient and DOE-2 model algorithm was used for the exterior.

Internal gains (i.e., occupation profile and lighting) correspond to the ones established in Decree 141/2012 about the minimum conditions for building habitability. It was assumed that the module was occupied by 4 people. As previously indicated, the container is a simple shelter for staff rest. Thus, following the guidelines of EnergyPlus and ASHRAE [33], 72 W per person were assigned. Based on the usual regime of activities in deployments, it was established that these 4 people are relieved every 6 h, leaving one hour between relays for personal hygiene, meals, etc. For lighting, three compact DTT fluorescents of 18 W each were added. These lights will be on during the relay periods. The building infiltration was calculated using an empirical method based on the Spanish Technical Building Code (CTE) [34]. Container volume and facade, roof, door and window areas were considered to calculate infiltration, resulting 0.63 air changes per hour. The forced ventilation was included. A constant minimum ventilation flow rate of 4 s^−1^ was set according to the basic health document (DBHS) of the CTE specifications [29].

Set point temperatures range was fixed from 18 to 25 °C. This temperature range was established as per NATO regulations [30]. The meteorological data (input in the EnergyPlus simulation model) were downloaded from [35] in EPW format. As this type of container is going to be used in different locations, the thermal needs must be analyzed in in different scenarios. However, the potential of energy reduction is limited to the PCM melting temperature [25]. In this study case: EC-PCM40-BN10 (33.7 °C), EC-PCM40 (34.4 °C) and EC-PCM40-CNF2 (35.4 °C). Thus, the PCM effect was studied in arid and warm temperate climates. Six different locations were analyzed (Table 3). The time step employed for the CondFD algorithm calculations of the walls where the PCM layer was added was set to 1 min.

## 3. Results

### 3.1. Experimental

**Figure 3:**
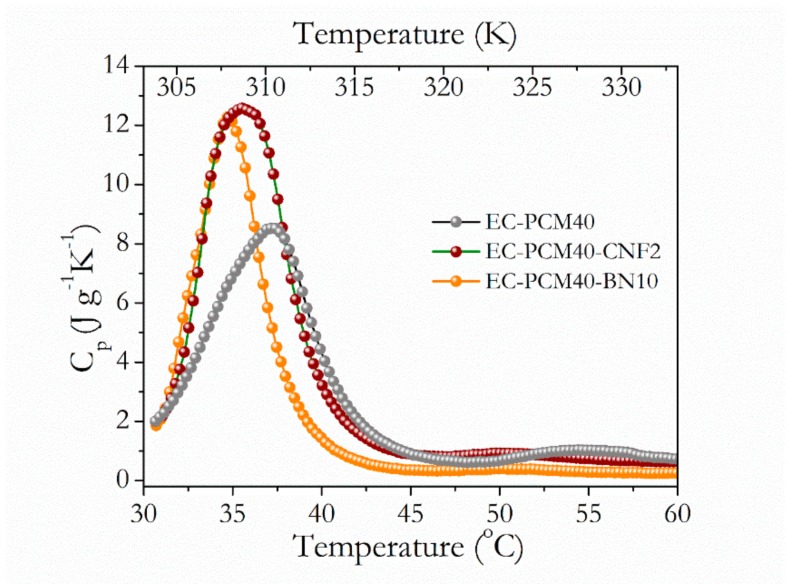
C_p_ versus Temperature (°C) curves of the EC-PCM40, EC-PCM40-CNF2, and EC-PCM40-BN10 formulations.

The thermal conductivity values of the samples are tabulated in Table 2, where conductivities of the EC-PCM40, EC-PCM40-CNF2, and EC-PCM40-BN10 were noted as 0.293, 0.303, and 0.415 W∙mK^−1^ [8], whereas the densities were estimated as 0.85, 0.84, and 1.03 g∙cm^−3^, respectively. The addition of the conductive agents resulted in higher thermal conductivity resulting in an enhancement of 3.3% and 41.6% for the CNF and BN filler, respectively, and the values are comparable with other reports for similar filler makeup [16]. Studies with poly (imide) matrix and 10 wt.% BN filler have reported a thermal conductivity of ~0.26 W·mK^−1^ [36], whereas paraffin matrix material and 10 wt.% BN nanosheets filler have reported a thermal conductivity of ~0.48 W·mK^−1^ [37]. For 2 wt.% CNF filler in a paraffin matrix, values of 0.411 W·mK^−1^ have been noted [38]. It is noteworthy that, for the current work, an epoxy resin was utilized as the support matrix for mitigating paraffin leakage, which was not employed in other studies [38]. The lower density of the composite with 2 wt.% CNF can be ascribed to the nanostructured natured of the CNFs. Figure 3 shows the Cp (T) curves of EC-PCM40, EC-PCM40-CNF2 and EC-PCM40-BN10 composite formulations. As seen from the graphs, peak transition temperatures were noted as 37.2 °C, 35.4 °C and 34.6 °C for the composites with no filler, 2 wt.% CNF and 10 wt.% BN, respectively. The addition of fillers resulted in lower peak transition temperatures.

### 3.2. EnergyPlus Simulation Results

Annual energy heating and cooling demands of the module and the achieved savings are shown in Table 4. Results show that the inclusion of any of the three configurations with PCM has a significant impact in arid and warm temperate climates. In terms of annual energy savings (adding heating and cooling), best results are obtained for Albuquerque (BSk), presenting a reduction per year of 483 kWh for EC-PCM40-CNF2, 476 kWh for EC-PCM40, and 470 kWh for EC-PCM40-BN10, which would be equivalent to 0.73 BOE per year [39] for the best-case scenario (EC-PCM40-CNF2). The fact that the PCM was selected for a specific location increased the savings for the Albuquerque location by more than 10% compared to that obtained in previous studies [25]. In other locations the use of PCM provided significant energy savings but was limited to heating or cooling periods. This is the case of Yuma and Tenerife, where the heating season is not representative in terms of cooling demands. Thus, when analyzing cooling demands, best PCM performance is shown for Yuma. In this location by including the EC-PCM40-CNF2, an annual energy saving of 411 kWh was achieved (Table 4). The melting range of the three PCM configurations (33.7 to 35.4 °C) is appropriate to reduce energy demands (both heating and cooling) in the selected locations. However, it is more useful to develop the PCM composite to reduce heating or cooling, as in this case. Thus, Yuma which is the location with the highest average temperature (23.9 °C), the energy savings achieved, in absolute terms, are much greater. On the other hand, Albuquerque, which has the lowest temperature, the energy savings could reduce from 24.9% to 26.6% during the heating season (Table 4). This fact shows that the PCM composite with lower melting temperature maximizes the savings during heating season. Thus, EC-PCM40 shows better results during heating season for all locations analyzed compared to the other two compositions with fillers.

**Figure 4:**
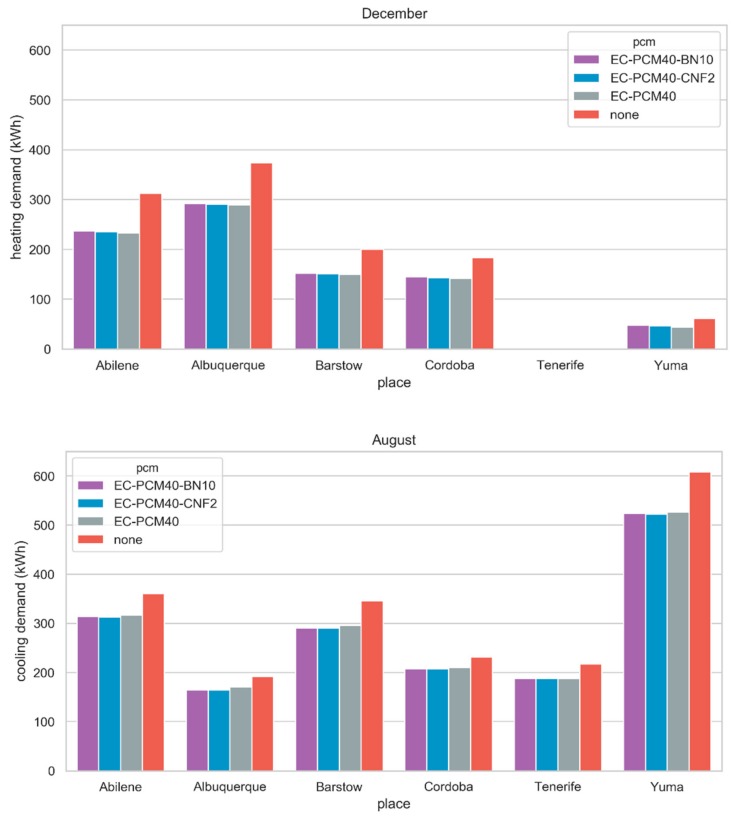
Heating and cooling demands for December and July for the six locations.

Figure 4 shows the heating and cooling demands for the months in which they are maximum in each of the locations. The trend shown in the figure is similar during all heating and cooling seasons. Although the difference between the different formulations of PCM is modest, the positive effect is evident. As these are warm climates, the highest consumption and savings, in absolute values, can be found in the cooling demands. It is important to note that even if two locations are classified as the same climate, different results can be found. This is due to the daily average temperature and the number of cycles (fusion-solidification) that the PCM can do. Both Albuquerque and Barstow are BSk (tropical and subtropical steppe climate). If both locations are studied in detail, the difference in altitudes or the HDD index make the climatic conditions in Albuquerque very different from Barstow (Table 3). Although the percentage of savings can be considered similar, the total energy demands are not the same (Table 4).

**Figure 5:**
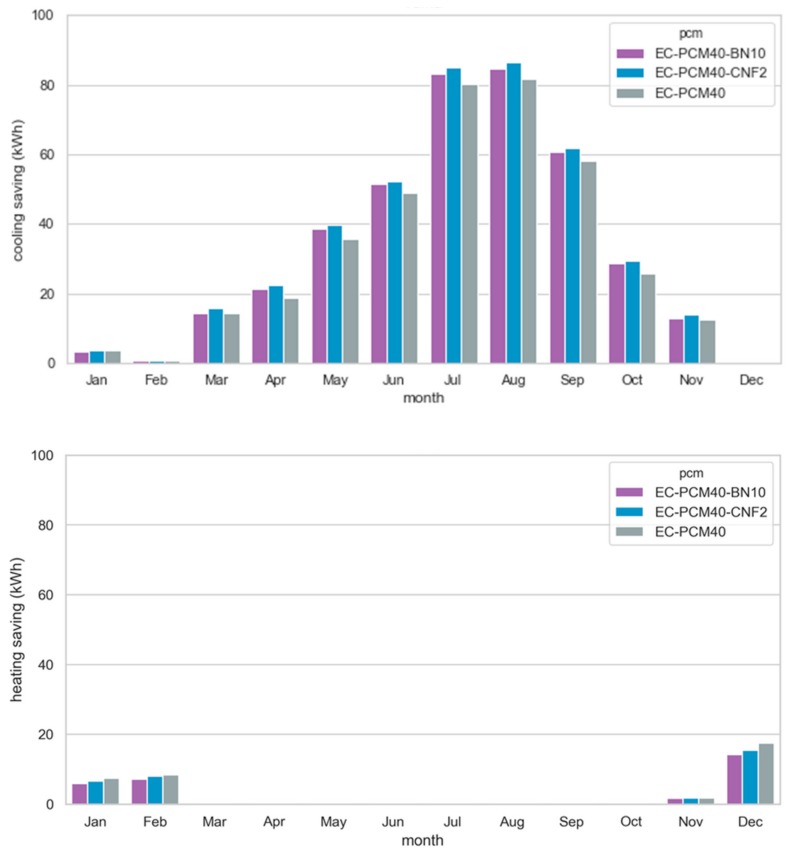
Monthly cooling and heating savings in Yuma overall a year.

Yuma (BWh) is the location where higher cooling demands are reached and, in absolute terms, higher savings are achieved. There is cooling and heating demand during the whole year and the inclusion of any PCM may reduce both loads during each month. However, there are differences between the three PCM formulations. That is, EC-PCM40 shows better performance during the heating season, while EC-PCM40-CNF2 shows the highest savings during cooling season (Figure 5).

**Figure 6:**
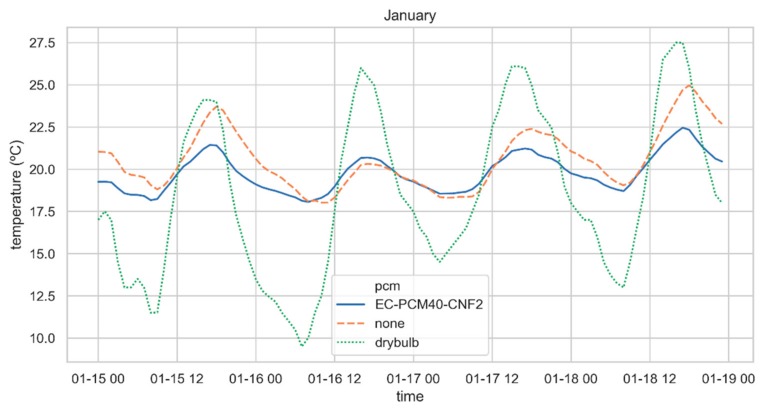
Indoor air temperature in case with EC-PCM40-CNF2 and without PCM and outside air drybulb temperature in Yuma during 72 h (from January 15th to January 17th) when there is not HVAC.

As previously indicated, shipping containers used as buildings have overheating problems because of irradiance, especially during cooling season (summer). The PCM layer addition to the envelope offers high thermal inertia. This produces a delay and a reduction in the peak heat load, which leads to a lower consumption of energy. Reducing the temperature of the indoor air in the cooling season is key to energy savings, as it has been shown that for each degree increase in temperature, the energy consumption is increased by approximately 7% [29]. Figure 6 shows the temperature of the air inside the container when there is no HVAC. In fact, the relocatable nature of the shipping containers when used as building could imply the absence of any HVAC system. The performance of a PCM enhanced container envelope depends on different factors: melting temperature, latent heat, and the external climatic conditions [40,41]. A PCM melting temperature outside the operational temperature values -dependent on climatic conditions- makes the PCM layer useless. In this case the PCM addition allows to increase the thermal mass in a narrow temperature range, resulting in energy savings. The same behavior as exhibited by EC-PCM40-CNF2 in Figure 6 was found for the other two PCM formulations.

**Figure 7:**
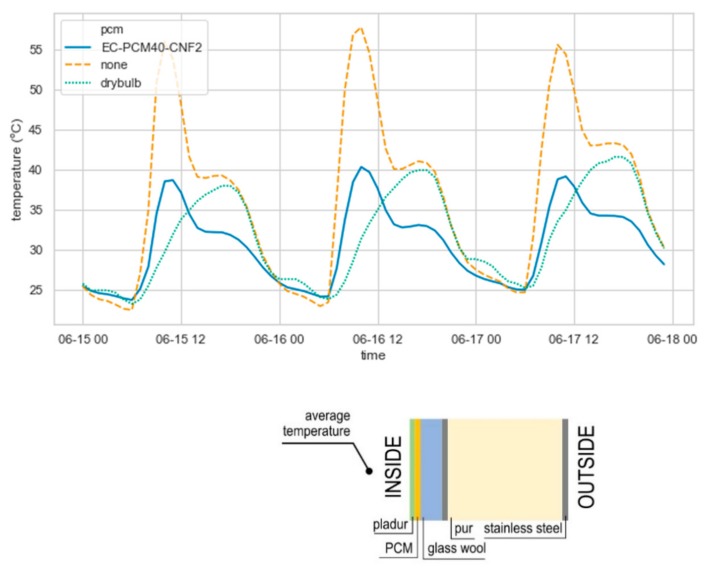
External wall temperature with EC-PCM40-CNF2 and without PCM (top) Outside air drybulb temperature in Yuma during 3 d (from January 15th to January 18th) (bottom).

To exhibit the impact of PCM layer in the envelope, the temperature of the interior side of south oriented wall was analyzed (Figure 7). Differences of more than 15 °C were observed between the wall with EC-PCM40-CNF2 layer and without PCM layer. Results shown a delay in the behavior of the wall with PCM layer, due to its higher thermal mass. A container envelope characterized by low thermal inertia leads to overheating air inside when affected by solar irradiance. The same pattern observed in Yuma (Figure 7) was found in the other locations. As expected, in summer months the PCM effect in the temperature inside the container is higher. The lower energy consumption results when adding PCM are due to the absorption and release of the heat load during the PCM transition phases (melting and solidification).

**Figure 8:**
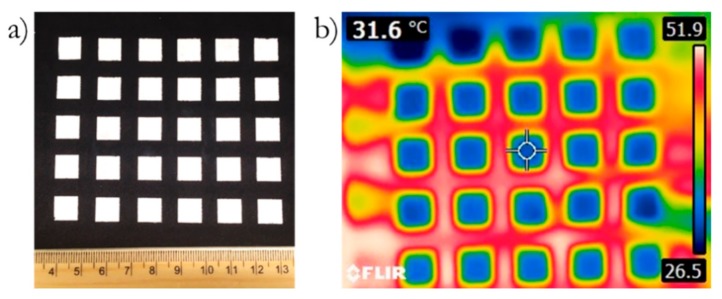
(**a**) An image showing EC-PCM40-BN10 formulation template casted on nylon; (**b**) thermal image of the sample showing temperatures close to 50 °C in the fabric alone while sections containing EC-PCM40-BN10 remain at 31 °C or less.

Given the differences in energy demands and savings at different times of the year and even in the same day by location, it seems adequate to consider the use of these materials in some type of plug and play architecture, such as a removable liner. Since the paraffin solidification process occurs as an exothermic process, liberating heat, it will be recommended that the liners are removed when the temperatures drop below the PCM peak transition temperature. Figure 8 shows a photograph of the template casted EC-PCM40-BN10 sample deposited on nylon fabric and corresponding thermal image taken with an infrared camera. It is evident from the thermal image that the PCM composite sample displays cooler localized temperature compared with the surrounding temperature, demonstrating the feasibility of material integration in flexible substrates as well as the envelopes already modeled.

## 4. Conclusions

This manuscript presents the successful integration of the thermal conductivities, densities, and latent heat determined in laboratory settings for novel epoxy-PCM formulations with a model that calculated the effects of their use in terms of energy demands, savings and temperature profiles of the interior and the walls of a portable shelter. The results indicated that all the Epoxy-PCM formulations employed could have a positive impact on the total energy savings (between 16% to 22%) for the locations selected.

The use of CNF and BN showed an increase in performance when compared with the formulation with no thermal filler additives. It is believed that the projected improvements are linked to the changes that the thermal enhancers produce in the formulation’s peak transition temperatures, thermal conductivities and latent heat.

The differences in climates for the locations selected rendered demands, savings and temperatures that varied up to 10% for the same formulation. 

Given the differences in energy demands and savings rendered by the modeling efforts for the heating and cooling cycles, it is recommended that the epoxy-PCM materials be used as removable liners or another form of plug and play architecture.

The formulations selected showed great potential to reduce the energy demands, increase savings, and result in more adequate temperatures for living and storage spaces applications.

## Figures and Tables

**Table 1:** Epoxy-PCM-Filler sample formulation components.

Sample	Filler	Epoxy (Resin + Hardener) (wt.%)	Carbopol (wt.%)	PCM (n-nonadecane) (wt.%)	Filler (wt.%)
EC-PCM40	None	55	5	40	0
EC-PCM40-CNF2	CNF	53	5	40	2
EC-PCM40-BN10	BN	45	5	40	10

**Table 2:** Construction details.

Category	Materials			
	Element	Conductivity (W/m/K)	Specific Heat (J/kg/K)	Layer Thickness (mm)
**External wall**	Stainless steel	17	460	0.5
	Polyurethane (PUR)	0.022	1400	250
	Stainless steel	17	460	0.5
	Insulation	0.03	800	63.5
	EC-PCM40	0.293	*	5.3
	EC-PCM40-CNF2	0.303	*	5.3
	EC-PCM40-BN10	0.415	*	5.3
**Ground**	Extruded polystyrene	0.034	1540	300
**Roof**	Stainless steel	17	460	0.5
	Glass wool	0.04	7955	63.5
	Plaster (ceiling))	0.25	1000	150
	Insulation	0.03	800	63.5
	EC-PCM40	0.293	*	5.3
	EC-PCM40-CNF2	0.303	*	5.3
	EC-PCM40-BN10	0.415	*	5.3
**Door**	Stainless steel	17	460	0.5
	Polystyrene	0.18	1500	250
	Stainless steel	17	460	0.5

* Data in experimental section.

**Table 3:** Locations description.

	Location Description	Köppen-Geiger Climate Classification	Elevation (ft)	Latitude	Longitude	Annual CCD	Annual HDD
**Abilene**	Texas, US	Cfa	1784	32°25’N	99°41’W	2410	2558
**Albuquerque**	New Mexico, US	BSk	5326	35°03’N	106°37’W	1322	4065
**Barstow**	California, US	BSk	232	34°54’N	117°1’W	2171	960
**Cordoba**	Andalusia, ESP	Csa	295	37°5’N	4°50’W	1624	2046
**Santa Cruz de Tenerife**	Canary Islands, ESP	Csb	151	28°27’N	16°15’W	1806	89
**Yuma**	Arizona, USs	BWh	207	32°39’N	114°36’W	3551	1148

* Note: CCD, cooling degree days; HDD, heating degree days.

**Table 4:** Heating and cooling demands for each location

	**Abilene**				**Albuquerque**			
	**No PCM**	**EC-PCM40**	**EC-PCM40-CNF2**	**EC-PCM40-BN10**	**No PCM**	**EC-PCM40**	**EC-PCM40-CNF2**	**EC-PCM40-BN10**
**Heating** **(kWh)**	962.76	707.98	717.30	725.04	1414.46	1038.69	1054.06	1062.48
**Cooling** **(kWh)**	1423.67	1211.62	1200.20	1208.07	684.32	583.80	561.29	566.61
**Savings heating** **(%)**		26.46	25.50	24.69		26.57	25.48	24.88
**Savings cooling** **(%)**		14.89	15.70	15.14		14.69	17.98	17.20
	**Barstow**				**Córdoba**			
	**No PCM**	**EC-PCM40**	**EC-PCM40-CNF2**	**EC-PCM40-BN10**	**No PCM**	**EC-PCM40**	**EC-PCM40-CNF2**	**EC-PCM40-BN10**
**Heating** **(kWh)**	560.82	403.39	412.35	417.96	633.31	480.04	487.76	494.50
**Cooling** **(kWh)**	1385.31	1164.17	1142.30	1149.42	716.42	639.06	632.56	637.31
**Savings heating** **(%)**		28.07	26.47	25.47		24.20	22.98	21.92
**Savings cooling** **(%)**		15.96	17.54	17.03		10.80	11.71	11.04
	**Santa Cruz de Tenerife**				**Yuma**			
	**No PCM**	**EC-PCM40**	**EC-PCM40-CNF2**	**EC-PCM40-BN10**	**No PCM**	**EC-PCM40**	**EC-PCM40-CNF2**	**EC-PCM40-BN10**
**Heating** **(kWh)**	0	0	0	0	109.17	74.30	77.52	80.42
**Cooling** **(kWh)**	877.37	717.68	725.38	734.63	2492.61	2112.28	2081.49	2092.71
**Savings heating** **(%)**		—	—	—		31.94	28.99	26.34
**Savings cooling** **(%)**		18.2%	17.3%	16.27		15.26	16.49	16.04
